# Complications and Mortality in Hospitalised Patients With Decompensated Cirrhosis of Liver in a Tertiary Care Centre in Nepal

**DOI:** 10.7759/cureus.9996

**Published:** 2020-08-24

**Authors:** Subash Bhattarai

**Affiliations:** 1 Gastroenterology, Manipal College of Medical Sciences, Pokhara, NPL

**Keywords:** complications, liver cirrhosis, mortality, predictors

## Abstract

Background

Patients with decompensated cirrhosis present with various complications and are associated with increased inpatients mortality. This study aimed to evaluate the complications and mortality in hospitalised patients with decompensated cirrhosis of liver.

Methods

This descriptive, cross-sectional, hospital-based study included 754 decompensated cirrhotic patients. The primary endpoints were mortality and hospital stay. The data analysis was done using Statistical Product and Service Solutions (SPSS) version 20 (IBM Corp., Armonk, NY). The chi-square test was used to compare the differences between different predictors of mortality with p<0.05 considered significant.

Results

A total of 754 patients (mean age 54±11.51 years; male/female ratio of 3.6:1) were studied. Ascites was the most common complication (99.2%) followed by upper gastrointestinal (UGI) bleed (42.3%), hepatic encephalopathy (32.5%), rebleeding (33.2%), spontaneous bacterial peritonitis (26%), and hepatorenal syndrome (19.1%). Inpatient mortality was 19.8%. The most common causes of mortality were rebleeding (21.5%) followed by hepatic encephalopathy (HE) (18.7%), hepatorenal syndrome (HRS) (14.7%), and spontaneous bacterial peritonitis (SBP) (12.1%). The presence of Grades IV HE, the presentation with shock, Child Turcotte Pugh (CTP) C, rebleeding, variceal bleed, HRS, hyponatremia (<130 mEq/L), the requirement of ≥3 units of blood and blood products, co-existence of hepatocellular carcinoma (HCC), and multiple comorbidities and complications in a single patient were strong predictors of mortality (p≤0.05).

Conclusions

Ascites followed by UGI bleed, hepatic encephalopathy, rebleeding, spontaneous bacterial peritonitis, and hepatorenal syndrome were common complications among the admitted decompensated cirrhotic patients. Inpatient mortality was high. The most common cause of mortality was rebleeding followed by hepatic encephalopathy, HRS, and SBP.

## Introduction

Liver cirrhosis is a progressive chronic liver disease. Histologically, it is characterized by diffuse, fibrosing and nodular condition that disrupts the normal architecture of the liver [1]. The main aetiological causes are excessive alcohol consumption, chronic viral hepatitis B and C, obesity, and nonalcoholic fatty liver disease. The clinical presentation of cirrhosis varies with the aetiology. Clinical features of cirrhosis are secondary to portal hypertension and/or hepatocellular injury. Many times, patients may present with severe liver injury without any obvious clinical signs [2]. Cirrhosis is classified into two stages: compensated and decom­pensated. Decompensated cirrhosis has either jaundice or varied complications like splenomegaly, ascites, spontaneous bacterial peritonitis, hepatic encephalopathy, development of esophagogastric varices, and variceal bleed [1]. Liver cirrhosis is an important health problem worldwide and is associated with increased morbidity and mortality. About 80% of patients with newly diagnosed hepatocellular carcinoma (HCC) have preexisting cirrhosis [3].

Liver cirrhosis is a common disease in Nepal, especially with easy accessibility and very common use of alcohol. National data on decompensated cirrhosis and complications in hospitalized patients are scarce. This research was undertaken to study the various complications and assessments of mortality among hospitalised patients with decompensated liver cirrhosis admitted at Manipal College of Medical Sciences and Teaching Hospital, a tertiary care hospital in Gandaki Province, Nepal.

## Materials and methods

This descriptive, cross-sectional, hospital-based study was carried out in the unit of Medical Gastroenterology under the Medicine department at Manipal College of Medical Sciences and Teaching Hospital, Nepal from January 2018 to June 2020 over a period of 30 months. The study was approved by the Institutional Review (MEMG/IRC/374/GA). Informed consent was obtained from patients or patient relatives.

All cases with jaundice or ascites or any other clinical features suggestive of decompensated cirrhosis of liver admitted in the ICU and/or ward admitted under unit of Medical Gastroenterology at Manipal Teaching Hospital were included in the study. Child Turcotte Pugh (CTP A, B or C) scoring system was used to assess the severity and prognosis whereas West Haven Classification (Grade I to IV) was used for grading of hepatic encephalopathy. Stable cirrhotic patients presenting to outpatient department (OPD), patients with compensated cirrhosis, acute fulminant hepatitis or those with non-cirrhotic portal hypertension, those with incomplete records, and those who fail to give consent were excluded from the study.

The sample size was collected using the formula:

Sample size: Z^2^ x [p x (1-p)] /e^2^

Z: 1.96 (critical value of the normal distribution for 95% confidence interval)

`p: sample proportion (prevalence of the disease or 0.5 if no prevalence is known)

e: standard error (0.05) or when prevalence is given, 20% of prevalence

The minimum sample size required and calculated as per the equation with no known prevalence of cirrhosis; 95% CI (Z=1.96, α=0.05, and assumed p=0.5, q=0.5) was 384.

Data regarding demographic variables, varied presentation, and complications at admission were documented. Blood investigations like complete blood count, platelet count, blood grouping, electrolytes, liver function test, prothrombin time/international normalized ratio (PT/INR), coagulation profile, tumour marker (alpha fetoprotein ) for HCC and viral serologies were collected. Ultrasonography and computed tomography (CT) scan of the abdomen were done for assessment of liver echogenecity and to rule out hepatocellular carcinoma, collateral vessels, and evidence of other features of portal hypertension and other complications. Upper GI endoscopy was done for screening of varices and evaluation of upper GI bleed. Therapeutic endoscopic variceal band ligations were performed with bleeding and/or large varices. Clinical outcomes of these decompensated liver cirrhotic patients during hospitalization including mortality were studied.

Data analysis and statistical methods

Data were collected on a structured proforma. All categorical data were expressed in percent and absolute number. All numerical continuous data were expressed in mean±SD. Chi-squared test was used to test for significant difference of proportions (categorical data). All tests were analyzed with a 95% confidence interval and a p-value of <0.05 was considered statistically significant. The data analysis was done using Statistical Product and Service Solutions (SPSS) version 20 (IBM Corp., Armonk, NY).

## Results

A total of 796 patients were screened for study eligibility. However, 25 patients were taken away to home or elsewhere by relatives against medical advice despite initial management and few days of admission, and 17 were excluded because of inadequate data. Finally a total of 754 patients comprising 590 (78.2%) male and 164 (21.8%) female cirrhotic subjects were eligible for the study (M:F=3.6:1).

The mean age of subjects was 54±11.51 years with a range of 24-85 years of age. Patients were further classified as per sex and age groups as in Table 1 with maximum cases in 50-69 years of age group.

**Table 1:** Age groups/sex distribution of patients under study

Sex	Age Groups				Total
	<35 Yrs	35-49 yrs	50-64 yrs	≥65 yrs	
Male	66	165	281	78	590
Female	33	49	70	12	164
Total	99	214	351	90	754

Alcohol-related chronic liver disease was the most common aetiology of cirrhosis in 671 patients (89%). Chronic viral hepatitis-related cirrhosis was seen in 67 (8.9%) patients (chronic Hep B 5.7% and chronic Hep C 3.2%). The remaining 16 (2.1%) cases were classified as cryptogenic. Four hundred and sixty (61%) patients were documented smokers. Cirrhotic subjects were classified according to CTP classes. The majority, 596 (79%), were of Child class C and 158 (21%) patients were of Child class B.

These subjects presented with varied clinical presentations. The most common presentation was abdominal distension (n=754, 100%) followed by anorexia (n=740, 98.1%), fatigue (n=720, 95.5%), and vomiting (n=640, 84.9%). Icterus (n=712, 94.4%) followed by pallor (n=686, 90.9%), pedal edema (n=612, 81.2%), and loss of body hair (n=560, 74.3%) were the common signs. One hundred and eighty-six (24.7%) patients presented with shock.

Many of the subjects presented with varied complications of liver cirrhosis. Ascites was commonest and seen in 748 (99.2%) patients. Five hundred and nine (67.5%) presented with gross and tense ascites. Repeated large-volume paracentesis was required in 510 (67.6%) patients. Three hundred and nineteen (42.3%) patients presented with UGI bleed. Ruptured varices were the aetiology in 145 (45.5%) cirrhotics. Rebleeding was seen in 106 (33.2%) patients. This was followed by hepatic encephalopathy in 245 (32.5%), spontaneous bacterial peritonitis (SBP) in 196 (26%), and hepatorenal syndrome (HRS) in 144 (19.1%). The majority (n=174) of the patients with hepatic encephalopathy were in HE grade II/III. Twenty-six patients were in hepatic encephalopathy Grade IV. Forty-one (5.4%) cirrhotic subjects were also diagnosed with hepatocellular carcinoma (HCC).

The mean hospital stay of the patients was 6.3 days. One hundred and forty-nine (19.8%) had inpatient mortality within 14 days. The most common causes of mortality were rebleeding (21.5%) followed by hepatic encephalopathy (18.7%), HRS (14.7%), SBP (12.1%), and sepsis (10.7%). Deaths due to pneumonia and coronary artery diseases were also common (Table 2).

**Table 2:** Causes of death in patients with decompensated cirrhosis

Complications	Number	Percentage (%)
Rebleeding	32	21.5
Hepatic encephalopathy	28	18.7
Hepatorenal syndrome (HRS)	22	14.7
Spontaneous Bacterial Peritonitis (SBP)	18	12.1
Sepsis	16	10.7
Pneumonia	15	10.1
Coronary artery disease	12	8.1
Others	6	4.1
Total	149	100

The presence of HE of Grades IV, the presentation with shock, CTP C, rebleeding, variceal bleed, HRS, hyponatremia (<130 mEq/L), the requirement of ≥3 units of blood and blood products, co-existence of HCC, multiple comorbidities, and complications in a single patient were significant predictors of increased mortality (p≤0.05; Table 3).

**Table 3:** Predictors of mortality in patients with decompensated cirrhosis HE: hepatic encephalopathy, SBP: spontaneous bacterial peritonitis, CTP: Child Turcotte Pugh, HCC: hepatocellular carcinoma

Parameters	Discharged	Mortality	P-value
Grade IV HE	0	26	<0.001
Shock (SBP <90 mmHg)	124	62	0.001
CTP C	467	134	0.001
Variceal bleed	117	28	0.05
Rebleeding	74	32	<0.001
HCC	18	23	<0.001
≥2 complications	128	88	<0.001
Repeated Large volume paracentesis	395	115	0.05
Blood transfusion ≥3 units	66	33	0.002
≥2 co morbidities	256	106	0.001
Hyponatremia (<130 mEq/L)	144	32	0.002

## Discussion

In our study comprising 754 decompensated cirrhotics, 590 (78.2%) were male and 164 (21.8%) were female (M:F=3.6:1). The mean age of subjects was 54±11.51 years. The mean ages of cirrhotics are 48±8 years and 45.8±10.45 years in Rai et al. and Bhattacharyya et al., respectively [4,5]. Hajiani et al., Maskey et al., and Khan et al. demonstrated male predominance with mean ages of 47, 49, and 57.5 years, respectively [6-8].

A majorities (79%) was of Child class C and 21% of patients were of Child class B in the current study. Whereas, Rai et al. and Hajiani et al. in their studies reported 50% and 51% of patients with Child class C, respectively [4,6].

Ascites was the most common complication and was seen in 99.2% of patients in the current study. This was followed by UGI bleed (42.3%), hepatic encephalopathy (32.5%), rebleeding (33.2%), spontaneous bacterial peritonitis (26%), hepatorenal syndrome (19.1%), and HCC (5.4%). The common complications according to Bhattacharyya et al. are ascites (78.6%), variceal bleeding (43.4%), hepatic encephalopathy (21.6%), SBP (4.2%), HRS (2.7%), HCC (1.3%), hypersplenism (0.4%), and sepsis (12.8%) [5]. Ascites followed by HE, UGI bleed, and HRS were common complications in the study by Rai et al. [4]. The prevalence of SBP was 24.7% and 34.9% in hospitalized patients by Syed et al. and Jain et al., respectively [9,10].

Inpatient mortality was seen in 19.8% in the current study. It was 19.1% and almost similar in the study by Pathak et al. in a previous Nepalese study [11]. The most common causes of mortality were rebleeding (21.5%) followed by hepatic encephalopathy (18.7%), HRS (14.7%), and SBP (12.1%) in the current study. Similar were the findings in the study by Bhattacharyya et al. [5]. The most common cause of mortality was rebleeding (47.8%) followed by hepatic encephalopathy (39.1%) and hepatorenal syndrome (13.1%) in the study by Bhattacharyya et al. [5]. Whereas, hepatic encephalopathy (72.2%) was the most common cause of deaths among cirrhotics in the study by Pathak et al. followed by variceal bleeding (33.3%) and hepatorenal syndrome (33.3%) [11].

The presence of HE of Grades IV, the presentation with shock, CTP C, rebleeding, variceal bleed, HRS, hyponatremia (<130 mEq/L), the requirement of ≥3 units of blood and blood products, co-existence of HCC, multiple comorbidities, and complications in a single patient were strong and significant predictors of increased mortality in the current study. Variceal bleed, hyponatremia, HRS, SBP, CTP C, and HE were similarly associated with increased mortality in the study by Rai et al. [4].

Mortality was 100% in patients with hepatic encephalopathy grade IV in the current study. Bajaj et al. in their study have also stated that severe HE was an independent predictor of mortality [12].

## Conclusions

Ascites was the most common complication followed by UGI bleed, hepatic encephalopathy, rebleeding, spontaneous bacterial peritonitis, and hepatorenal syndrome. Inpatient mortality was high. The most common causes of mortality were rebleeding followed by hepatic encephalopathy, HRS, and SBP. The presence of HE of Grades IV, the presentation with shock, CTP C, rebleeding, variceal bleed, HRS, hyponatremia, co-existence of HCC, multiple comorbidities, and complications in a single patient were strong predictors of mortality in patients with decompensated cirrhosis.
